# Requirement of PEA3 for Transcriptional Activation of FAK Gene in Tumor Metastasis

**DOI:** 10.1371/journal.pone.0079336

**Published:** 2013-11-18

**Authors:** Shufeng Li, Xiaofeng Huang, Dapeng Zhang, Qilai Huang, Guoshun Pei, Lixiang Wang, Wenhui Jiang, Qingang Hu, Renxiang Tan, Zi-Chun Hua

**Affiliations:** 1 The State Key Laboratory of Pharmaceutical Biotechnology, College of Life Science and School of Stomatology, Affiliated Stomatological Hospital, Nanjing University, Nanjing, People’s Republic of China; 2 Changzhou High-Tech Research Institute of Nanjing University and Jiangsu TargetPharma Laboratories Inc., Changzhou, People’s Republic of China; 3 The State Key Laboratory of Quality Research in Chinese Medicine, Macau University of Science and Technology, Macau, People’s Republic of China; Institut de Génétique et Développement de Rennes, France

## Abstract

Focal adhesion kinase (FAK) is a non-receptor tyrosine kinase critically involved in cancer metastasis. We found an elevation of FAK expression in highly metastatic melanoma B16F10 cells compared with its less metastatic partner B16F1 cells. Down-regulation of the FAK expression by either small interfering RNA or dominant negative FAK (FAK Related Non-Kinase, FRNK) inhibited the B16F10 cell migration *in vitro* and invasiveness *in vivo*. The mechanism by which FAK activity is up-regulated in highly metastatic cells remains unclear. In this study, we reported for the first time that one of the Est family proteins, PEA3, is able to transactivate FAK expression through binding to the promoter region of FAK. We identified a PEA3-binding site between nucleotides −170 and +43 in the FAK promoter that was critical for the responsiveness to PEA3. A stronger affinity of PEA3 to this region contributed to the elevation of FAK expression in B16F10 cells. Both *in vitro* and *in vivo* knockdown of PEA3 gene successfully mimicked the cell migration and invasiveness as that induced by FAK down-regulation. The activation of the well-known upstream of PEA3, such as epidermal growth factor, JNK, and ERK can also induce FAK expression. Furthermore, in the metastatic human clinic tumor specimens from the patients with human primary oral squamous cell carcinoma, we observed a strong positive correlation among PEA3, FAK, and carcinoma metastasis. Taking together, we hypothesized that PEA3 might play an essential role in the activation of the FAK gene during tumor metastasis.

## Introduction

Focal adhesion kinase (FAK) is a cytoplasmic tyrosine kinase that plays critical roles in integrin-mediated signal transductions and also participates in growth factor receptor signaling [1]–[3]. Extensive studies indicated that FAK is an important mediator of cell adhesion, growth proliferation, survival, angiogenesis and migration [4]–[5]. FAK-mediated signaling largely depends on its kinase activity and associated protein–protein interactions. For example, after integrin clustering, FAK binds to the cytoplasmic tail of β-integrin and results in phosphorylation of FAK tyrosine Y397 [6]–[7]. Such auto-phosphorylation of Y397 generates a high-affinity binding site for Src. The interaction with Src results in ERK ^MAPK^ activation to initiate proliferation [8]–[9]. Once further phosphorylated by Src, FAK can also recruit Jun N-terminal kinase (JNK) to focal adhesion sites and the JNK pathway is also implicated in promoting FAK-initiated signals controlling tumor cell invasion [10]–[11].

Studies have shown that normal tissues have low expression of FAK, while primary and metastatic tumors significantly overexpress this protein [12]–[13]. Previously, NF-κB and p53 were reported as important mediators for transcriptional regulation of FAK gene in several human cell lines [14] and N-MYC is involved in regulating FAK expression in human neuroblastoma [15]. In many tumors, the levels of FAK correlate with their relative degree of invasiveness [11]. In our previously reported results, we showed that down-regulation of the FAK expression by either small interfering RNA or dominant negative FAK (FAK Related Non-Kinase, FRNK) inhibited the B16F10 cell migration *in vitro* and invasiveness *in vivo* [16]. However, the mechanism by which FAK activity is differentially up-regulated in highly metastatic cells is still unknown.

PEA3/E1AF belongs to the ETS family proteins, a characteristic feature of this transcription factor family proteins is that they share an evolutionarily conserved ETS domain, which mediates the binding to purine-rich DNA sequence containing a central GGAA/T core consensus and additional flanking nucleotides [17]–[18]. Many ETS family proteins, like ETS-1, ETS-2 or PEA3 are downstream nuclear targets of the signal transduction cascades [19].

In this study, we tried to determine which transcription factor was preferentially involved in the FAK gene upregulation in highly metastatic melanoma cancer cells. Differential FAK expression was detected in B16F10 and B16F1 cells, two melanoma cell sublines with different metastatic potentials. Our results indicated that the up-regulation of FAK in highly metastatic cells was mediated by PEA3 on the FAK promoter. Furthermore, a positive correlation among PEA3, FAK, and tumor metastasis in human clinic tumor specimens was also observed. The present research might offer a novel mechanism for the regulation of FAK gene during tumor metastasis.

## Materials and Methods

### Cell Lines and Transfection

B16F10 and B16F1 melanoma cells were purchased from ATCC. Cell transfection was performed with Lipofectamine transfection reagent (Invitrogen, USA). Unless otherwise indicated, cells were harvested at 48 h after transfection.

### Plasmids

The Myc-tagged E1AF expression vector E1AF-pcDNA3.1 was kindly provided by Dr. Jianxin Gu (Shanghai Medical College of Fudan University, China). Mouse pEVRF-ETS-1 expressing vector was a gift from Dr. B. J. Graves (University of Utah. USA). Mouse pRK-HA-ETS-2 expressing vector was a gift from Dr. K.E.Boulukos (Université de Nice, France). pSRalpha-HA-JNK1 was a gift from Dr. Roger Davis (University of Massachusetts Medical School, USA). pcDNA-neo/HA-MAPK was a gift from Dr. Jacques Pouyssegur (University of Nice-Sophia Antipolis, France). Mouse FAK RNA interference vector psiFAK was constructed by our laboratory previously [16]. RNA interference plasmid was constructed using the pRNAT-U6.1/Neo vector (GenScript, USA). RNA interference target sequences were selected from the mouse PEA3 sequence (GenBank accession number: NM_008815). Each candidate target sequence was analyzed by BLAST search to ensure that the hit would be unique to the E1AF/PEA3 mRNA. Target oligo nucleotides were synthesized (5-GATCCCGTAAAGGCACTGCTCTCCATGGTTGATATCCGCCATGGAGAGCAGTGCCTTTATTTTTTCCAAA-3; 5-AGCTTTTGGAAAAAATAAAGGCACTGCTCTCCATGGCGGATATCAACCATGGAGAGCAGTGCCTTTACGG-3), annealed, and cloned into pRNAT-U6.1/Neo vector between the Bam HI and Hind III sites. A negative control vector comprising a scrambled sequence was also prepared as mock. The pRNAT-U6.1/Hygro/siFluc vector producing siRNA against luciferase was used as an unrelated siRNA control (Genescript, USA).

### 5′RACE

Total RNA from the mouse B16F10 cells was extracted by Trizol kit (Invitrogen, USA). A proportion of the RNA was subjected to 5′-RACE using BD SMART RACE cDNA Amplification kit (Clontech, USA). First round PCR was performed with the gene-specific antisense primer P1 (5′- CTTTAATACTCGTTCCATTGCACC-3′) and the second round PCR employed the gene-specific antisense primer P2 (5′ -GCCAGTACCCAGGTGAGTCTTAGTA-3′). PCR products that contained the transcription initiation site were T-A cloned with pGEM-T vector (Promega, USA) and processed for sequencing.

### Preparation of Promoter Deletion Constructs

A 885 bp fragment (nucleotides from −842 to +43) of mouse *FAK* promoter was prepared by PCR amplification of mouse genomic DNA of NIH3T3 cells using a sense primer containing Kpn1 restriction site and an antisense primer containing a SmaI restriction site. Primers were synthesized on the basis of the reported genomic sequence for mouse FAK, forward 5′-CGTGGTACCATATGGCAAAATCTCGACAACTCA-3′ and reverse 5′-TATCCCGGGCCTCAGCGCAGAGCTCTAC-3′. Following digestion with restriction enzymes, the FAK promoter fragment was directionally cloned into the pGL3-Basic firefly luciferase expression vector (Promega, USA) to generate a “full-length” FAK reporter construct, and the correct insertion was confirmed by sequencing. The construct containing the truncated fragment −842/−356 was produced by digestion the −842/+43 fragment with Kpn I and BgL II then cloned into the vector pGL3-basic. Reporter genes containing sequentially truncated fragments (−554/+43, −255/+43, −170/+43, −55/+43) of the *FAK* promoter region were prepared in a similar manner using different sense primers containing Kpn1 restriction sites and the same antisense primer that was used for the full-length FAK reporter construction. Constructs containing different fragments of the mouse FAK promoter (−842/+256, −554/+256, −255/+256, −170/+256, −55/+256, +28/+256) were prepared in a similar manner as above with different sense primers containing Kpn1 restriction sites and another downstream antisense primer containing a Sma1 restriction site. All constructs were sequenced and confirmed. To prepare mutated promoters, the putative ETS transcription factor-binding site between nucleotide positions −82 and −88 was deleted and named p-170/+43 m. The mutation was created from p-170/+43 by PCR using Takara MutanBEST mutagenesis kit (Takara, Japan). Mutated constructs were sequenced, and the correct ones were selected for further experiments.

### Dual Luciferase Assay for Promoter Activity

Dual luciferase assay was performed as described previously with some modifications [14]. In brief, cells were plated on 24-well plates, cultured overnight and transfected using Lipofectamine transfection agent (Invitrogen, USA) according to the manufacturer’s protocol. For normalization of luciferase activity, the pRL-TK control vector encoding Renilla luciferase was used for co-transfection together with pGL3 plasmids. In some experiments, the pGL3-control vector was used in transfection as a positive control of promoter activity. This control vector contains an SV40 promoter plus enhancer sequences resulting in strong expression of luciferase gene in many types of mammalian cells. For all experiments, cells were cultured for 24 h after transfection and lysed with the Passive Lysis Buffer (Promega, USA). Lysates were analyzed using Dual-Luciferase Reporter Assay System kit (Promega, USA). Luminescence was measured on luminometer (Turner Biosystems Instrument, USA). All experiments were performed at least three times.

### RT-PCR

Total cellular RNA was reverse transcribed into cDNA by using AMV Reverse Transcriptase (Takara, Japan) with oligo (dT)_18_ as a primer. Equal amounts of the cDNA products were used as templates for subsequent PCR amplification. The oligonucleotide primers sequences were as follows: FAK sense, 5′-ACTCATCGAGAGATCGAGATGG-3′, antisense, 5′-GCCCTAGCATTTTCAGTCTTGC-3′; actin sense, 5′-GAAATCGTGCGTGACATCAAAG-3′, antisense, 5′-TGTAGTTTCATGGATGCCACAG-3.

### Northern Blot Analysis

Total cellular RNA was prepared using Trizol reagent (invitrogen, USA) according to the manufacturer’s instructions. Twenty micrograms of total RNA was electrophoresed through a denaturing gel and blotted onto a positively charged nylon membrane. After cross-linking with 120 mJ/cm^2^ ultraviolet irradiation, the filter was pre-hybridized in ExpressHyb™ hybridization solution (BD Biosciences-Clontech, USA). Hybridization was carried out with [32 p] labeled probe derived from PCR fragment of mouse FAK cDNA. The probe from PCR fragment of mouse actin cDNA was used as an internal control. The probe was labeled using a Prime-A-Gene random primer labeling kit (Promega, USA) according to the manufacturer’s instructions. The blotted membranes were washed and exposed to storage Phosphor screen overnight (Amersham Biosciences, USA).

### Electrophoretic Mobility-shift Assays (EMSA)

Binding studies were performed with double-stranded oligonucleotides containing the PEA3-binding site of *FAK* promoter p-170/+43 (sense oligonucleotide: 5′-CCACCTCGTCATCCCGGAACAGCGCTATCCG-3′). The oligonucleotides were annealed and end-labeled at the 5′ end with [*γ* -32 p] ATP and T4 polynucleotide kinase. The binding reaction was performed by preincubating 9 *µ*g of nuclear extract in 10 mM Hepes, pH 7.5, 50 mM KCl, 5 mM MgCl_2_, 0.5 mM EDTA, 1 mM dithiothreitol, 12.5% (v/v) glycerol and 2 *µ*g of poly(dI-dC) · (dI-dC) in a final volume of 20 *µ*l for 10 min at room temperature. Then the probe was added to the reaction mixture and incubated for an additional 30 min at room temperature. Competition experiments using a 100-fold molar excess of ETS-1/E1AF consensus (sense oligonucleotide 5′-GATCTCGAGCAGGAAGTTCGA-3′), WT (wild-type) or mutated (sense oligonucleotide: 5′-GGGCCACCTCGTCATC(Δ7)AGCGCTATCCGCGG-3′) unlabelled probe were performed. Supershift experiments were performed by adding 2 *µ*l of anti-PEA3 antibody to the reaction mixture together with the labeled probe. Samples were run on a 6% non-denaturating polyacrylamide gel (200 V for 3 h at 4°C) with 0.5× TBE (44.5 mM Tris, pH 8.3, 44.5 mM boric acid, 1.25 mM EDTA) buffer. Gels were dried and autoradiographed.

### Preparation of Nuclear Extracts and Western Blot Analysis

Nuclear extracts were prepared with Nuclear and Cytoplasmic Extraction reagents (NE-PER) (Pierce, USA), according to the manufacturer’s protocol. A total of 30 µg of protein from each sample was electrophoresed by 10% SDS-PAGE and transferred to PVDF membrane. After blocking with TBS containing 5% nonfat milk and 0.1% Tween-20 for 2 h, the membrane was incubated with the primary antibody at 4°C overnight. After washing with TBS containing 0.1% Tween-20 three times, each for 5 min, the membrane was then incubated with horseradish peroxidase (HRP)-labeled secondary antibody for 2 h at room temperature. The membrane was then developed by using the enhanced chemiluminescent (ECL) detection systems.

### Invasion and Migration Analyses

Cell migration assay was performed using Transwell inserts as previously described with some modifications [16]. In brief, the under surface of the membrane was coated with fibronectin (10 µg/ml) in PBS (pH7.4) at 37°C for 2 h. The lower chamber was filled with 0.6 ml of 10% FBS supplemented with DMEM medium. Before experiment, cells were serum–starved overnight (DMEM plus 0.5%BSA), then re-suspended in migration medium (DMEM plus 0.5%BSA), 1×10^6^ cells in a volume of 0.1 ml were added to the upper chamber. After incubation at 37°C for 12 h, cells on the upper surface of the membrane were removed. The migrant cells attached to the lower surface were fixed in 10% formalin and stained with a solution containing 1% crystal violet and 2% ethanol in 100 mmol/L borate buffer (pH9.0). The number of migrated cells on the lower surface of the membrane was counted under a microscope in 5 fields at a magnification of 100.

Wound healing assays were performed as previously described [16]. Briefly, cells plated onto fibronectin-coated (10 mg/ml) 24-well plate were transfected with plasmids, serum-starved (DMEM plus 0.5%BSA) overnight, wounded with a 200 µl pipette tip, washed with PBS, and incubated in medium containing 10% FBS. Migration of the wounded cells was visualized and quantified.

### Animal Model and in vivo Gene Transfer

C57BL/6J mice (6 to 8 weeks of age) were obtained from Shanghai Laboratory of Animal Center (Shanghai, China) and housed in a temperature-controlled sterile room where humidity and light were carefully monitored. Animal welfare and experimental procedures were performed strictly in accordance with high standard animal welfare and other related ethical regulations approved by Nanjing University. For s.c. footpad injections, 5×10^4^ cells in a volume of 20 µl were injected into the left hind footpads. Tumor volume was monitored by measurement of the two maximum perpendicular tumor diameters with calipers every other day. When tumors reached a size of ∼5×5 mm, the mice were arbitrarily assigned to different groups. 10 µg plasmids complexed with *in vivo*-jetPEI™ (Polyplus-transfection Inc. USA) at N/P ratio of 10 were injected into tumor for each animal per injection, and the injection were repeated every three days for a total of three times. Tumors were measured every other day and their volumes were calculated. For metastasis assays, tumor-bearing mice were sacrificed at 17^th^ day after the first treatment, lungs and lymph nodes were removed for H&E staining analysis.

### Tail Vein Metastasis Assay

To produce experimental metastasis, the C57BL/6J mice were injected intravenously with 10^6^ cells in 0.2 ml of PBS via tail vein. After 20 days, the mice were euthanized, and their lungs were resected and photos were taken (Nikon Coolpix 4500, Japan) before fixation in Bouin’s solution for further analysis. The numbers of metastatic nodules on the surface of the organs were counted macroscopically.

### Ethical Issues and Patient Samples

Ethical approval was obtained from Ethics Committee of Affiliated stomatological hospital, medical school of Nanjing University; Written consent was taken from patients as approved by the committee. Archival formalin-fixed paraffin-embedded human oral squamous cell carcinoma specimens were collected.

### Immunohistochemistry

Five-micron sections of paraffin embedded tissue were immunostained with primary monoclonal antibodies against PEA3 (SC-113, 1∶40 dilution; Santa Cruz, USA) and FAK (BD Biosciences Pharmingen, USA). The sections were examined microscopically by two pathologists who were blinded to the clinicopathological characteristics and patients’ outcome. Nuclear expression of E1AF and cytoplasmic expression of FAK were defined as positive when immunoreactivity was observed in >10% of cancer cells of the tumor.

### Statistics and Presentation of Data

The results were expressed as mean ± SEM. The statistical analysis involving two groups was performed by means of Student’s *t*-test, whereas analysis of variance (ANOVA) followed by Dunnett’s multiple comparison test was used in order to compare more than two groups. All data were processed with SPSS 10.0 software.

## Results

### Highly Metastatic B16F10 Cells have Higher FAK Expression Level than that of Low Metastatic B16 F1 Cells

**Figure 1:**
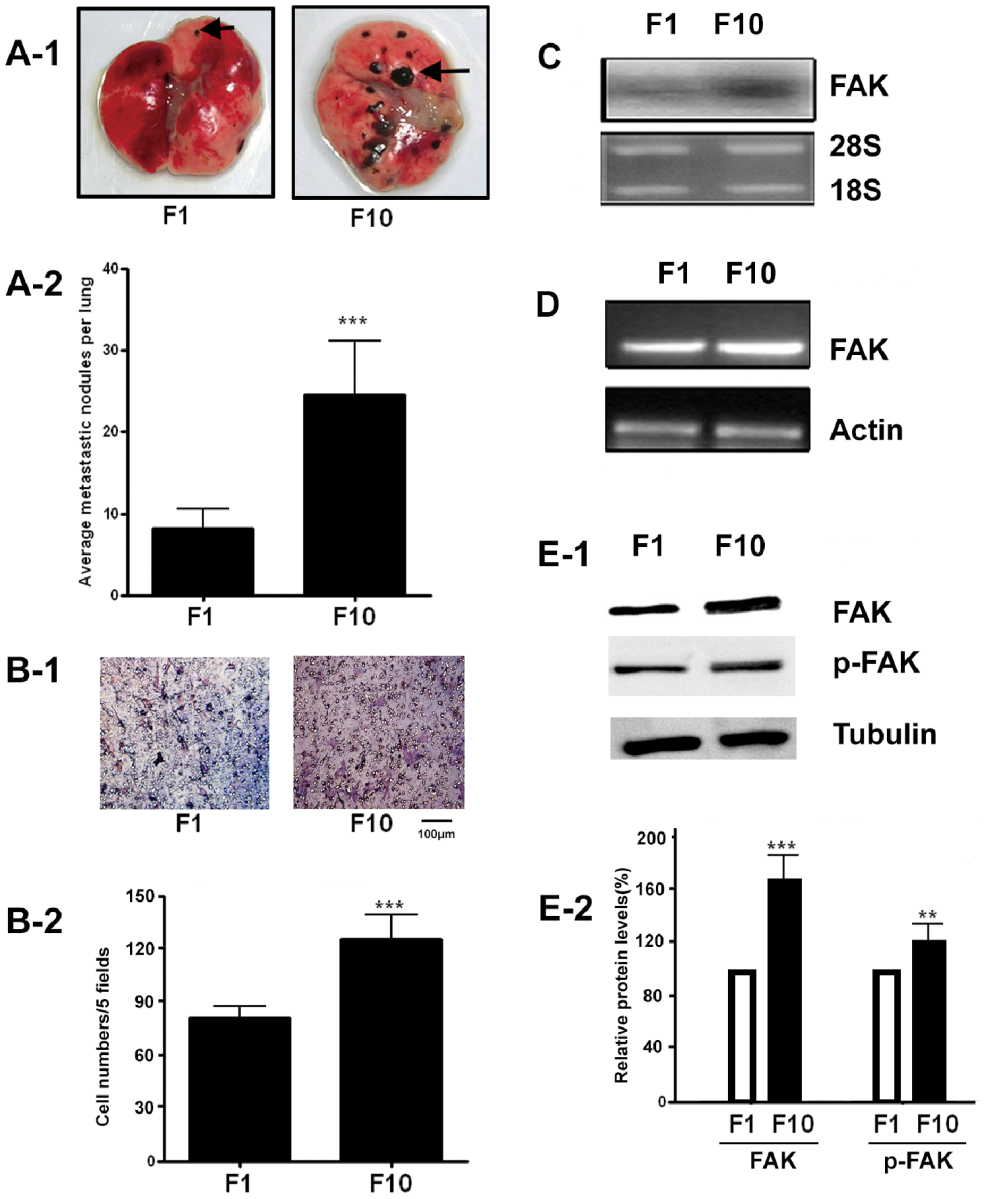
Increased FAK expression in highly metastatic B16F10 melanoma cells. (A–B) Comparison of the *in vivo* and *in vitro* metastatic potential of B16F10 and B16F1 melanoma cells. B16F10 and B16F1 cells (1×10^6^) were injected intravenously into C57BL/6J mice via tail vein. After 20 days, lungs from the mice were resected and analyzed for metastasis. The representative lung in mice injected with F1 or F10 cells are shown (A-1). After fixation in Bouin’s solution, metastatic nodules found on lung surface are counted and averaged (A-2). Data are presented as mean ± SEM of five mice. ***P<0.01. (B-1) Migration of F10 and F1 in vitro. F1 and F10 cells were incubated in the transwell insert with 10% FBS in the lower chamber for 12 h. Photo show representative membrane attached migrated cells. Scale bar = 100 µm. Quantitative analysis of the number of the cells migrated to the down side of the membrane (B-2). Data are mean ±SEM of three independent experiments. ***P<0.01. (C–D) Northern blot and RT-PCR analyses of FAK mRNA in B16F10 and B16F1 cells. β-actin was amplified in RT-PCR and used as an internal control. (E-1) Western blot analysis of FAK expression level and p-FAK level in B16F10 and B16F1 cells. Whole cell lysates from B16F10 and B16F1 cells were blotted with antibodies to FAK, phosphorylated FAK (Y397 p-FAK) or tubulin as a control. (E-2) Densitometric quantification revealed a significant increase in expression of FAK protein in F10 cell line. Densitometric units were normalized to Tubulin and then divided by F1 group results. (n = 3; ***P<0.01, **p<0.05 ).

**Figure 2:**
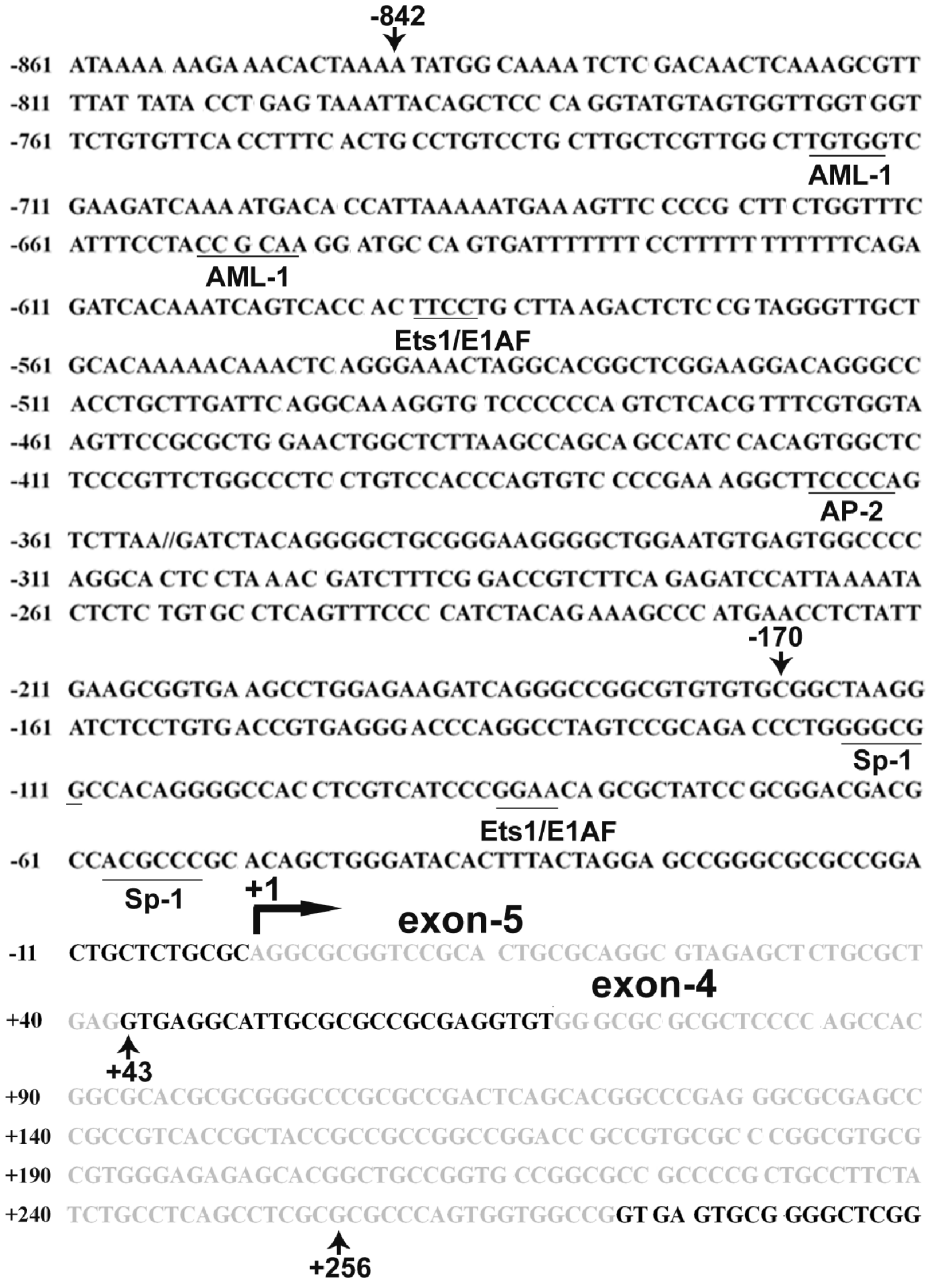
Schematic diagram of mouse FAK promoter region as well as putative transacting factor binding sites. This figure shows the genomic sequence (FAK genomic sequence from mouse RefSeq: NT_039621) which includes exon-5 and exon-4 of the FAK gene (in grey shadow). The sequence contains −861 bp genomic region immediately upstream of the FAK gene transcription start site (+1) and downstream of the +290 bp sequence. The position of the transcription initiation site was defined as +1 determined by us with 5′ RACE in the B16F10 cells and indicated with black bent arrow. This putative TATA box-less promoter contains GC-rich sequence. The putative binding sites for transcription factors Sp1, E1AF, AP-2 and AML-1 predicted by TRANSFAC program are underlined and labeled.

B16F1 and B16F10 cells are two mouse metastatic melanoma cell lines isolated from the parent cell line B16F0 by clonal selection of metastatic tumors. However, B16F10 cells have much stronger lung-colonizing potential than B16F1. When B16F1 and B16F10 cells were intravenously injected into mice, a significant increase of lung metastatic nodules were developed in mice injected with B16F10 cells than that in mice injected with B16F1 cells (Fig. 1A-1&Fig. 1A-2,***P<0.001). The in vitro migration results also showed that B16F10 cells were more motile than B16F1 cells (Fig. 1B-1 & Fig. 1B-2, ***P<0.001). FAK mRNA or protein level in B16F10 cells was also significantly higher than that in B16F1 cells (Fig. 1C, Fig. 1D, Fig. 1E-1 and Fig. 1E-2). We used these two melanoma cell lines (B16F1, B16F10) to characterize their invasive potential in vivo and in vitro, and measured the FAK gene expression level as a marker for invasion. Our results are consistent with the previously reported data [20], [21]. The cellular features of migration and invasion in vitro are nicely correlated with the well documented biological behavior of these cell lines to form secondary tumors in vivo at different rates in lung.

### Identification of a Novel First Exon by Mapping Transcription Start Site of the FAK Gene

To map the 5′ end of the mouse FAK gene, 5′-RACE was utilized on total RNA from B16F10 cells. The resulting PCR products were T-A cloned into pGEM-T vector (Promega) and processed for DNA sequencing. By comparing the DNA sequences of the RACE products with the previously reported mouse FAK leader sequences, we found the existence of two first exons. One was exon-4, as previously reported [22]. Another first exon, as shown in Fig. 2 and Supplementary Fig.S1, was discovered in our current study. We designated this first exon as exon-5. Correspondingly, the 5′ end of exon-5 (transcription start site) was set as +1. The two exons (−4 and −5) are mutually exclusive and both are spliced to exon-3. Both exon-5 and exon-4 have extremely high GC content (exon-5∶71.43%, exon-4∶81.86%). Meanwhile, the distance between exon-5 and -4 was rather small, suggesting that these two first exons may share the same promoter.

### Identification of the FAK Gene Promoter

**Figure 3:**
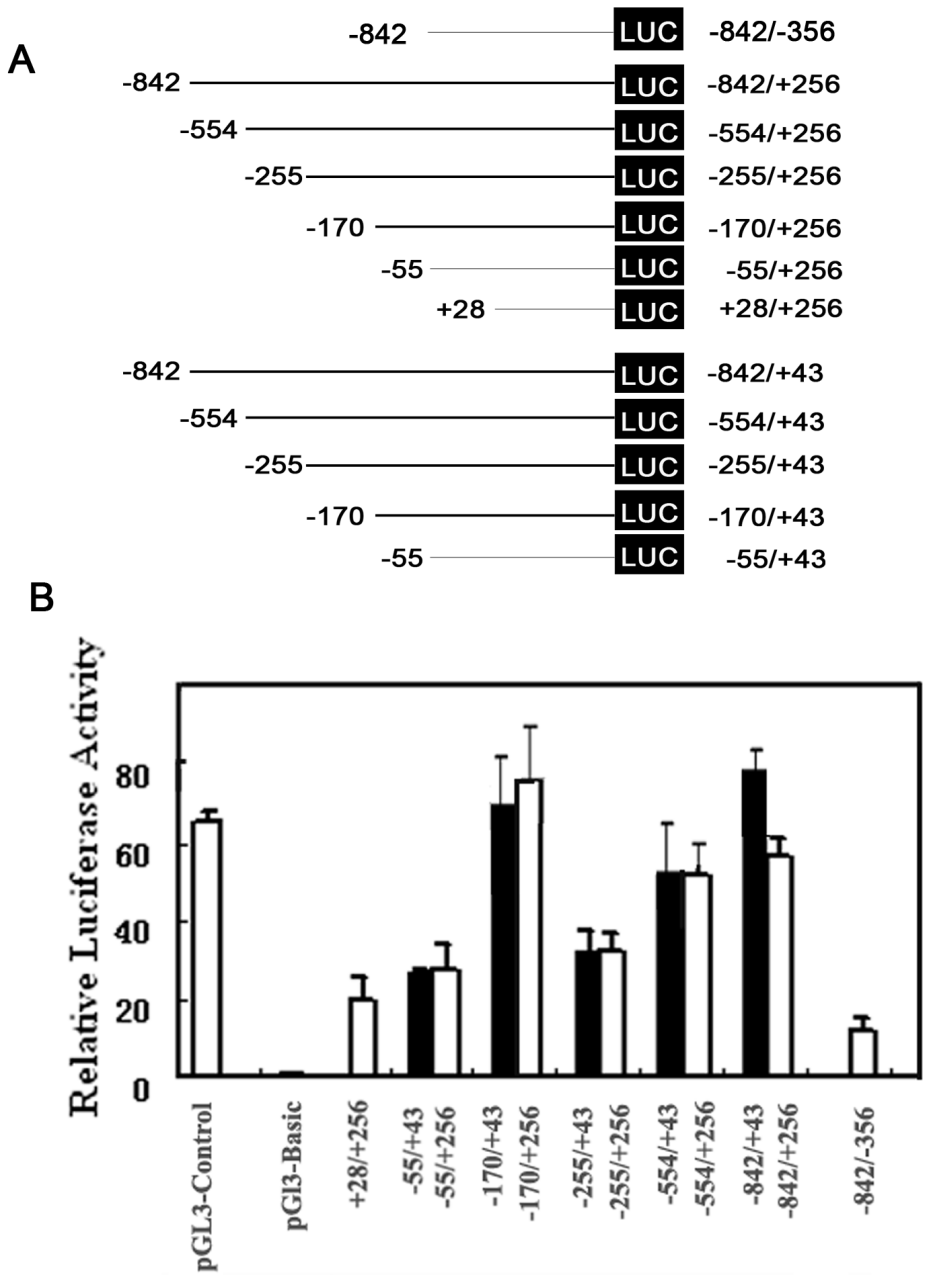
Progression deletion analysis of mouse FAK promoter. (A) Deletion constructs were cloned into the promoter-less pGL3-Basic vector. Different constructs with different deletions of the promoter fragments are shown. Numbers indicate 5′ and 3′ ends relative to the first transcription initiation site in each construct. (B) Identification of the minimal promoter for the FAK gene basal transcription. Luciferase plasmids harboring various lengths of FAK promoter regions were transiently transfected into B16F10 cells along with Renilla expressing plasmid. The luciferase activity was normalized to the internal Renilla control and standardized to the normalized activity from pGL3-Basic. Each value is the mean ±S.D. of at least three independent experiments.

To identify the mouse FAK gene promoter, we generated a series of deleted constructs carrying a promoter-Luc reporter gene. As shown in Fig. 3A and Fig. 3B, the longest construct p-842/+256 containing exon-5 and part of exon-4 displayed very high promoter activity in B16F10 cells. However, the promoter activities were reduced in truncated constructs, p-554/+256 and p-255/+256. Interestingly, further deleted construct p-170/+256 still had very high promoter activity. However, the promoter activities in other deleted segments, −55/+256 and +28/+256, were decreased dramatically. The promoter activity in the 3′-truncated fragment −842/−356 was abolished almost completely. These results indicated that the sequence between −170 and +256 are critical for basal FAK gene transcription.

To further minimize the core promoter region, additional 3′- deleted constructs were generated from position +256 to +43. The resultant p-842/+43, p-554/+43, p-255/+43, p-170/+43 and p-55/+43 constructs containing exon-5, but not exon-4 showed the similar promoter activities with that of p-842/+256, p-554/+256, p-255/+256 and p-170/+256. These results suggested that exon-4 and exon-5 may be transcribed from the same promoter, but from different initiation sites. Meanwhile, nucleotides from −170 to +43 consist of the minimal mouse FAK promoter that is responsible for FAK basal transcription.

### Identification of E1AF/PEA3 as a Major Trans-acting Factor Involved in the Regulation of FAK Gene Transcription

**Figure 4:**
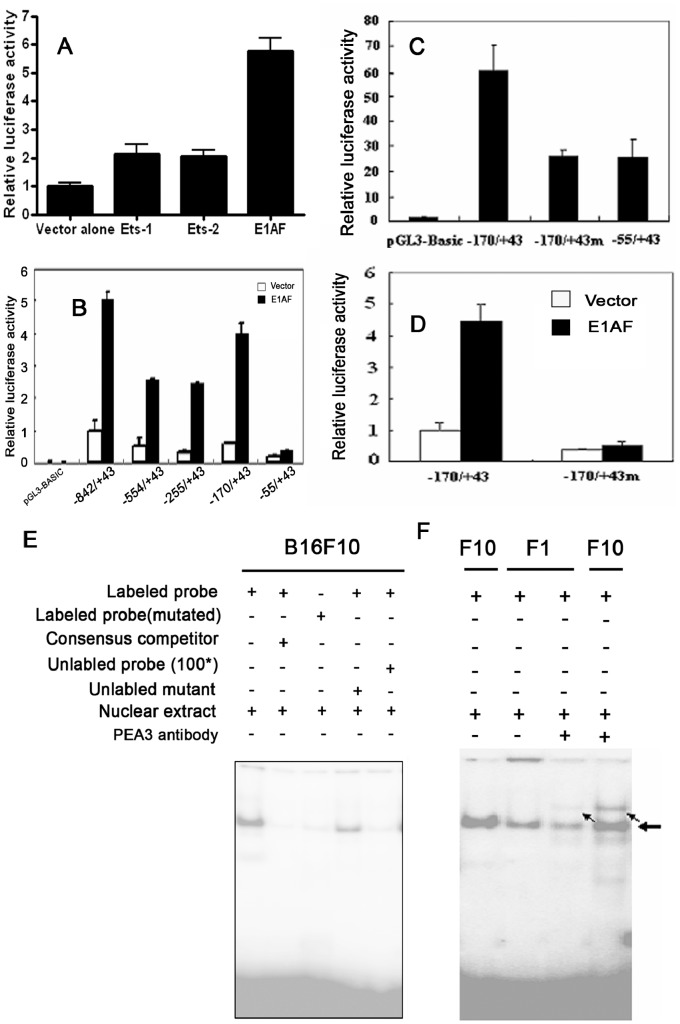
E1AF involved in the mouse FAK gene transcription activation in B16F1 cells. (A) Activation of FAK promoter by ETS transcription factor E1AF. The B16F1 cells were transfected with 1 µg plasmids expressing ETS-1, ETS-2, E1AF or mock plasmid along with 1 µg p-842/+43-luc and Renilla expressing plasmid. The luciferase activity of each sample was normalized to the internal Renilla control. Then the normalized luciferase activity was standardized to that of p-842/+43-luc with vector alone. Each value is the mean ±S.D. of at least three independent experiments. (B) Mapping the regions of the FAK promoter necessary for E1AF responsiveness. The B16F1 cells were transfected with p-842/+43-luc construct or the truncated FAK promoter constructs shown above and with or without E1AF expression vector. Luciferase activity was normalized to Renilla luciferase activity and standardized to the normalized activity from p-842/+43-luc with control vector alone. Data shown are the means ±S.D. of at least three independent experiments. (C) Site-directed mutation analysis of the FAK promoter. Cells were transfected with 2 µg p-170/+43-luc, p-55/+43-luc, or p-170/+43-luc devoid of E1AF binding site (p-170/+43 m) along with Renilla expressing plasmid. FAK promoter activity in B16F1 cells was decreased in the absence of E1AF binding site. (D) Stimulatory effect of E1AF overexpression on FAK promoter activation was prevented in the absence of E1AF binding site. B16F1 cells were co-transfected with 1 µg E1AF expressing plasmid, or empty vector along with 1 µg wide type p-170/+43-luc or mutated p-170/+43 m-luc and Renilla expressing plasmid. Luciferase activity of B16F1 cells co-transfected with empty vector and wide type p-170-luc was arbitrarily set at 1. E. EMSA was performed using nuclear proteins of B16F10 cells and the ^32^P-labeled mouse FAK promoter E1AF element probe. F. EMSA of the same amounts nuclear extracts from B16F10 cells and B16F1 cells incubated with ^32^P -labeled E1AF element probe and supershift by PEA3 antibody. The arrows indicate the supershifted bands.

Through computational analysis, several putative ETS transcriptional factors binding sites were predicted in the FAK promoter (Fig. 2). All ETS family factors share a conserved DBD of ∼85 amino acids. E1AF, a member of the PEA3 subfamily of ETS oncogenes, is a human homologue of mouse PEA3. To confirm the computational prediction, B16F1 cells were transfected with vectors expressing ETS family members, such as ETS-1, ETS-2 or E1AF in combination with p-842/+43-luc (Fig. 4A). The results revealed that the strongest activation of FAK promoter was obtained by overexpression of E1AF in B16F1 cells, whereas ETS-1 and ETS-2 only showed moderate stimulating effects.

To define functionally important cis-elements in the FAK promoter region that contributes to FAK promoter activation by E1AF/PEA3, B16F1 cells were co-transfected with the E1AF expression vector and FAK promoter variants (Fig. 4B). Luciferase assays showed that a deletion from −170 to -55 resulted in the loss of E1AF activation when comparing the p-170/+43-luc with p-55/+43-luc constructs. The minimal inducible promoter activity is located within the −170/+43 region of the FAK promoter. To further confirm the requirement of E1AF/PEA3 for FAK transcription, we deleted the putative E1AF/PEA3 binding site from p-170/+43-luc construct (170/+43 m) and found its luciferase activity was reduced to almost the same level as that of p-55/+43-luc promoter activity (Fig. 4C). Furthermore, overexpression of E1AF failed to stimulate the FAK promoter activity devoid of E1AF/PEA3 binding site (Fig. 4D).

To examine whether the increased FAK transcription was a result of enhanced E1AF/PEA3 binding to promoter region, EMSA experiments were performed. As shown in Fig. 4E, a DNA-protein complex was detected when the nuclear extracts of B16F10 cells were incubated with the double-stranded oligonucleotide probe containing PEA3 binding site. This DNA-protein complex was prevented in competition experiments. To compare the E1AF/PEA3 binding between B16F10 and B16F1 cells, same amount of nuclear extracts from these two cells were simultaneously incubated with labeled probe. As shown in Fig. 4F, the increased DNA-protein complex formation was detected in B16F10 nuclear extracts as compared to that detected with nuclear extracts from B16F1 cells. The intensity of the super shifted band from the B16F10 cells was also much higher than that of B16F1 cells, suggesting more PEA3 transcription factors binding to its promoter in B16F10 cells than in B16F1 cells.

### Knockdown of PEA3 Reduces the FAK Expression and Associated Metastatic Capacity of B16F10 Cells

**Figure 5:**
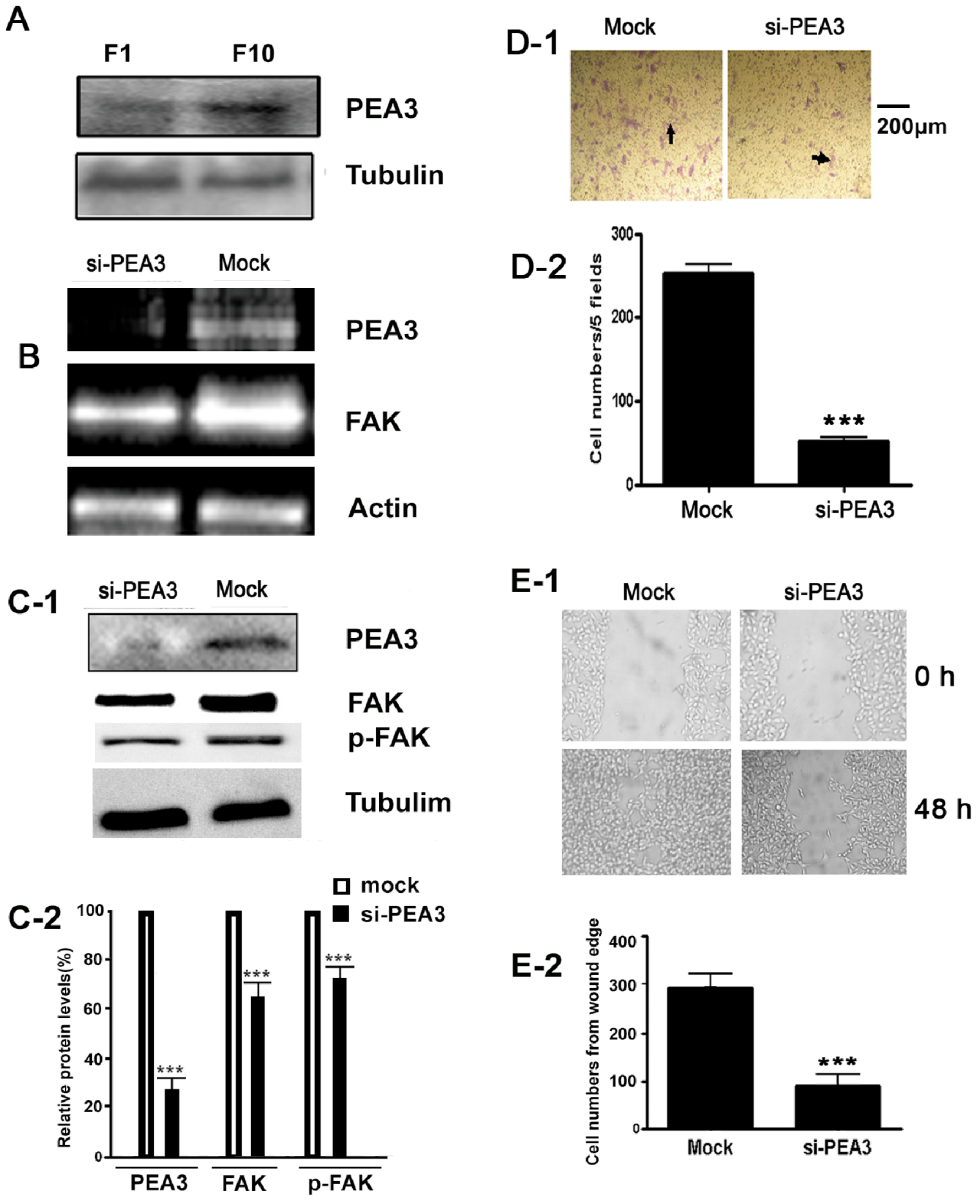
Involvement of PEA3 in B16F10 cells migration and invasion. (A) Elevated expression of PEA3 protein in nuclear extracts from B16F10 cells. PEA3 protein of each cell line nuclear extract was determined by western blotting using the anti-PEA3 antibody. Knockdown of PEA3 mRNA in B16F10 cells led to a reduction of FAK mRNA (B) or FAK protein (C). The B16F10 cells were transfected with 2 µg psi-PEA3 or mock plasmid for 48 h, RT-PCR analysis was performed to measure the PEA3 and FAK mRNAs (B), western blot analysis was performed to determine the PEA3,FAK and phosphorylated FAK (Y397 p-FAK) protein levels in the whole lysates of the B16F10 cells (C-1), tubulin or actin was used as a control. (C-2) Densitometric quantification analysis of western blot results in C-1. Densitometric units were normalized to tubulin and then divided by mock group results. (*n* = 3; ***, *p*<0.01) (D-E) Knockdown of PEA3 suppressed B16F10 cell invasiveness. After transfected B16F10 cells with mock or psi-PEA3 for 24 h, cells were collected and re-suspended in culture medium at a density of 1 × 10^6^ cells/ml. 100 µl of the cell suspension was plated into the upper wells of Transwell inserts containing 8 µm pore polycarbonate membranes pre-coated with fibronectin (10 µg/ml) on the under surface. The cells were allowed to migrate for 24 h at 37°C, then cells on upper wells were removed gently and those migrated to the under surface were fixed and stained. Representative membranes stained with crystal violet are shown (D-1), the arrows indicated the migrant cells. Scale bar = 200 µm. Quantitative analysis of the number of the cells migrated to the down side of the membrane (D-2). Data are mean ±SEM of three independent experiments. ***P<0.01, vs. mock control. (E) Knockdown of PEA3 in B16F10 cells suppressed the tumor cells migration. The B16F10 cells were transfected with si-PEA3 or mock vector. Then wound-healing scratch motility assays were performed in the fibronectin-coated plates in the presence of serum. Cell migration was assessed at 0 and 48 h. Representative images are shown (E1). Migration of cells into the wound was quantified (E-2). (*n* = 3; ***, *p*<0.01).

The above results raised the possibility that B16F10 cells might have higher PEA3 expression level than B16F1 cells. This hypothesis was confirmed by Western blot analysis (Fig. 5A). Since PEA3 plays a critical role in FAK transcription, successful knockdown of PEA3 mRNA level by siRNA against PEA3 resulted in a significant decrease in FAK mRNA level, as determined by RT-PCR analysis (Fig. 5B). Knockdown of PEA3 mRNA level also led to the down regulation of PEA3 as well as FAK protein expression in F10 cells (Fig. 5C-1 & Fig. 5C-2). To evaluate whether PEA3 is responsible for B16F10 cell metastasis phenotype, transwell migration assay was performed. The results, as shown in Fig. 5D-1 and Fig. 5D-2, demonstrated that knockdown of PEA3 expression in B16F10 cells resulted in a significant reduction of migration of B16F10 cells. Similarly, down regulation of PEA3 in B16F10 cells also resulted in fewer B16F10 cells to migrate into wound area as compared with mock plasmid transfected cells in wound healing experiments (Fig. 5E-1 & Fig. 5E-2).

### Intra-tumoral Delivery of siRNA against PEA3 or FAK Greatly Suppresses the Tumor Growth and Metastasis

**Figure 6:**
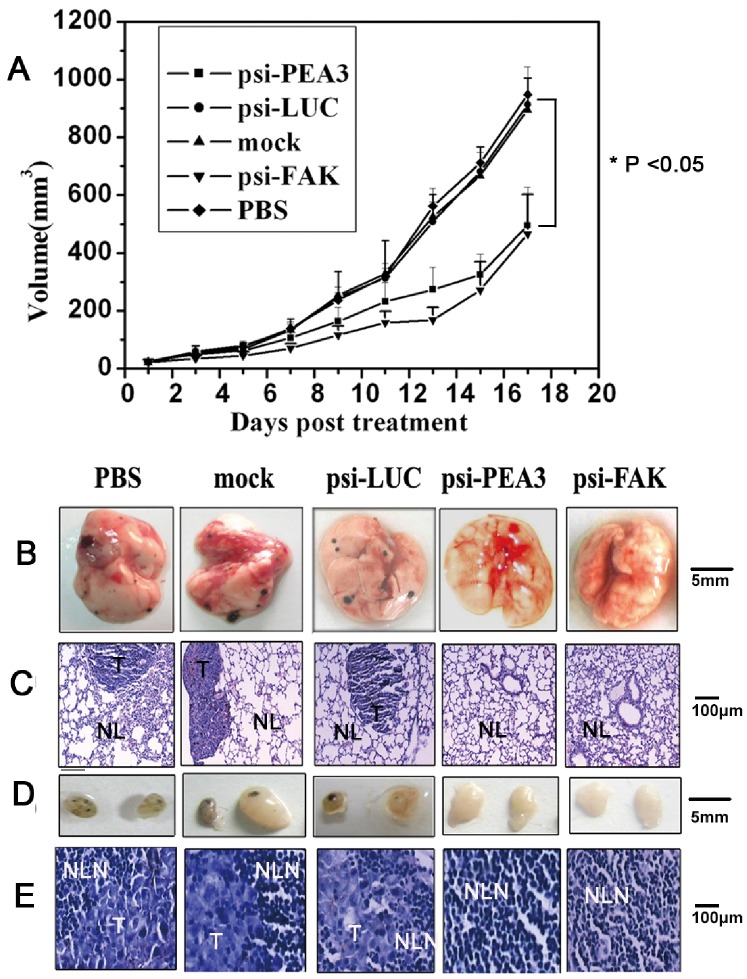
Intratumoral delivery of p-siFAK or p-siPEA3 inhibited cancer cell metastasis. (A) Intratumoral injection of p-siFAK or p-siPEA3 inhibited the solid tumor growth of implantable footpad model. Each group contains 8 mice. *P<0.05, as compared to control. 17 days post the first treatment, animals were sacrificed, and the lungs (B) as well as draining lymph nodes (D) were removed to examine the presence of any black metastatic foci. (C) Representative images (200×) of lung tissue sections stained with H&E. NL: normal lung tissue, T: metastasic tumor lesion. (E) Representative images (200×) of lymph node tissue sections stained with H&E, NLN: normal LN tissue, T: metastasic tumor lesion. Scale bars are included.

Next, we postulated that target delivery of siRNA against PEA3 into tumor in vivo will also delay the tumor growth and metastasis. As expected, when jetPEI-complexed plasmids were injected into tumors of C57BL6 mice, the solid tumor growth of groups treated with psi-FAK as well as psi-PEA3 were greatly suppressed as compared with other control groups (Fig. 6A). After experiment, the lungs as well as lymph nodes of tumor-bearing mice were removed. The results, as shown in Fig. 6B–6C revealed that multiple black metastatic foci were found on the lung surface of animals injected with PBS, mock plasmid and psi-LUC, whereas psi-FAK or psi-PEA3 injected groups remained unaffected. Similarly, in the isolated lymph nodes, black metastatic foci were found in groups injected with PBS, mock plasmid and psi-LUC, however, no obvious tumor nodules were identified in psi-FAK or psi-PEA3 injected animals (Fig. 6D& Fig. 6E, Table 1).

**Table 1:** Metastasis frequency of B16F10 cells in lymph nodes and lungs of tumor-bearing mice.

	LN metastases		Lung metastases	
Group	n	(%)	n	(%)
PBS	7/8	87.5%	4/8	50%
psi-LUC	6/8	75%	4/8	50%
psiFAK	0/8	0	0/8	0
psiPEA3	1/8	12.5%	0/8	0
Mock	7/8	87.5%	3/8	37.5%

### EGF Induced FAK Transcription

**Figure 7:**
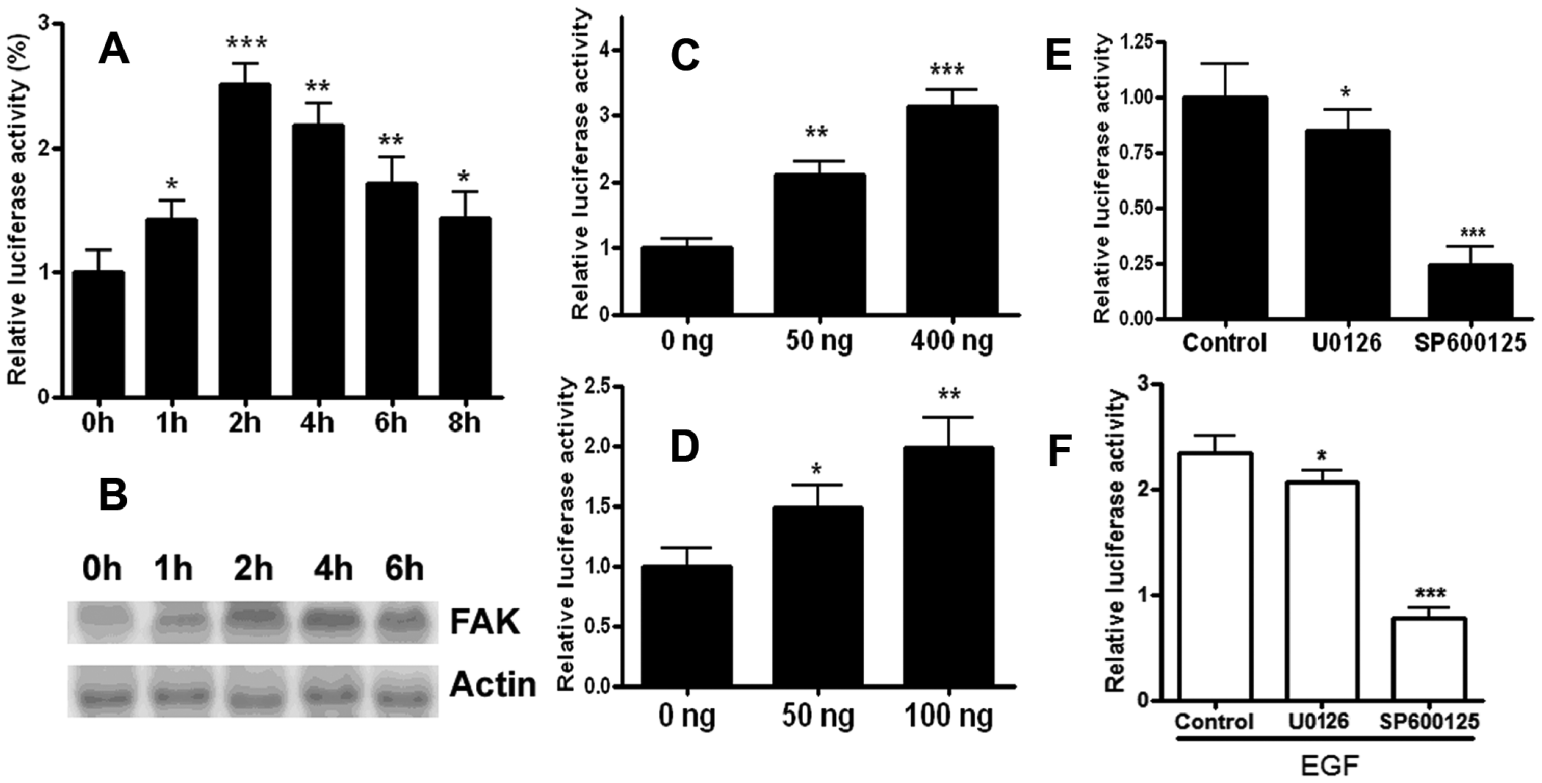
FAK gene expression can be induced by EGF. (A) Time-course of EGF stimulation on FAK gene transcription activation in B16F1 cells. The B16F1 cells were transfected with p-170/+43-luc construct along with Renilla expressing plasmid. After transfection, the cells were serum starved for 24 h, then pulsed with EGF (100 ng) for a designated period of time. Luciferase activity of transfected cells without EGF treatment was arbitrarily set at 1. Results shown are the means ±S.D. of six replicates. *P<0.05, **P<0.01, ***P<0.001 vs. control cells. (B) Time-dependent increase of the FAK mRNA level after EGF stimulation. The B16F1 cells were serum-starved for 24 h, and then pulsed with EGF (100 ng) for 1, 2, 4 or 6 h. After treatment, total RNA (30 µg) was separated, blotted and hybridized with labelled FAK cDNA probe, and actin probe as an internal control. Representative image of 3 independent experiment results is shown. (C-D) The B16F1 cells were transiently cotransfected with 0.5 µg p-170/+43-Luc plasmid and increasing amounts of plasmids expressing the JNK(C) or ERK (D). Luciferase activity of control cells without JNK or ERK overexpression was arbitrarily set at 1, *P<0.05, **P<0.01, ***P<0.001 vs control cells. (E) Effect of ERK or JNK inhibitor on FAK gene transcription activation in B16F10 cells. The B16F10 cells were transfected with p-170/+43-Luc plasmid, then treated with 20 µM SP600125 or U0126 for 6–8 h. *P<0.05, ***P<0.001 vs control cells. (F) Effect of ERK or JNK inhibitor on EGF-induced FAK gene transcription activation in B16F1 cells. The B16F1 cells were transfected with p-170/+43-Luc plasmid along with Renilla expressing plasmid. After serum starvation for 24 h, cells were pretreated with 20 µM SP600125 or U0126 for 0.5 h, pulsed with 100 ng EGF for an additional 3–4 h. *P<0.05, ***P<0.001 vs control cells.

PEA3 have been well defined as nuclear effectors of the Ras/MAPK signaling pathway, both ERK and JNK can regulate PEA3 activity [23]. We explored the possible relationship between Ras/MAPK and PEA3 in FAK gene transcription. As shown in Fig. 7A, EGF increased the FAK promoter activity (p-170/+43-luc) in a time-dependent manner which peaked at 2h after EGF treatment. FAK mRNA also increased in a time-dependent manner in serum-starved B16F1 cells following the addition of EGF (Fig. 7B), which corresponds well with the results obtained in FAK promoter studies (Fig. 7A). To investigate further the importance of MAPK in mediating the activity of FAK promoter, a series of transient transfections were performed (Fig. 7C & Fig. 7D). Transient overexpression of JNK and ERK in B16F1 cells resulted in a dose-dependent increase of FAK p-170/+43-Luc promoter activity (Fig. 7C & 7D). In contrast, the enhanced FAK p-170/+43-luc promoter luciferase activity in B16F10 cells was abolished by ERK and JNK inhibitors, U0126 and SP600125 (Fig. 7E). Furthermore, EGF-activated FAK transcription was also suppressed by pre-incubating with U0126 and SP600125 (Fig. 7F). All these results indicated the involvement of MAPK and PEA3 in FAK transcription activation in metastatic melanoma cells.

### FAK Protein Expression also Correlated Positively with PEA3 Expression in Specimens of Human Primary Oral Squamous Cell Carcinoma

**Figure 8:**
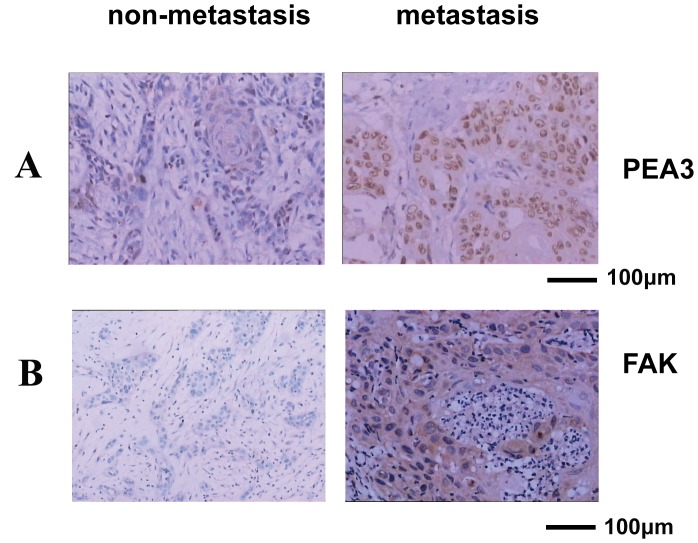
PEA3 and FAK were detected by the immunohistochemistry (IHC). Immunohistochemical analysis for PEA3 and FAK protein expression in paraffin wax embedded human primary oral squamous cell carcinoma specimen. Nuclear expression of PEA3 and cytoplasmic expression of FAK can be seen in representative cases of metastatic cancer tissue and are absent in some nonmetastasis carcinoma tissues. (original magnification, ×200). Scale bar = 100 µm.

To test what relationship between PEA3 and FAK in natural occurred cancer, we analyzed human clinic tumor specimens with human primary oral squamous cell carcinoma. We have shown that FAK expression was increased in metastasis oral squamous cell carcinoma compared to nonmetastasis samples (100 cancer samples from patients with stage I–IV oral squamous cell carcinoma. P<0.01) [supplementary Table S1, Fig. 8A and Fig. 8B]. A positive correlation between PEA3 and FAK expression in patients with high metastatic tumor was also observed (Fig. 8 and supplementary Table S2).

## Discussion

Metastasis is a complex process and the major cause of cancer morbidity. In this study, we have provided evidence that PEA3-induced FAK expression is necessary for melanoma cell migration and metastasis.

In our previous study, the invasive capacity of highly metastatic B16F10 cells was significantly reduced by decreasing FAK or introducing dominant negative FAK (FRNK) in B16F10 cells [16]. FAK has been implicated in tumor invasion and metastasis. But the mechanisms for the regulation of FAK expression in highly metastatic cancer cells have not been defined.

In this study, we first studied the 5-flanking region of the mouse FAK gene and identified the transcriptional start site and the minimal promoter region (−170/+43) of mouse FAK gene. Although it was predicted that FAK promoter region are not as highly conserved as the coding region between human and mouse [22], the mouse FAK promoter share common features with the previously reported human FAK promoter [14]. Both human and mouse promoters are GC-rich, lack typical TATA box.

We then investigated the involvement of Ets factors in the transcriptional regulation of FAK by comparing B16F10 with its less metastatic partner B16F1 cells. The Ets genes, which currently comprise nearly 30 members, encode transcription factors bearing conserved DNA binding domains (the ETS domain). PEA3/E1AF is believed to play an important role in tumor invasiveness and metastasis through transcription of metastasis-related genes including MMPs [24]–[27], HER-2/neu [28], cox-2 [29]–[30] and urokinase plasminogen activator [31]–[32]. Expression of PEA3/E1AF is also correlated with the metastasis phenotype of breast cancer [33]–[34] and invasive phenotype of neuroblastoma [35] and non-small-cell lung cancer [36]. In addition to PEA3/E1AF, other ETS family proteins like ETS-1 and ETS-2 have also been reported to be involved in tumor metastasis [37]–[39]. We found in this study that the expression of PEA3 was increased in highly metastatic melanoma cells compared with its low metastatic counterpart cells (Fig. 5A), which suggested that PEA3 might be involved in melanoma cell metastasis phenotype. Ets proteins are capable of regulating transcription by binding to the Ets-binding site (EBS) in the promoters of their target genes, and EBS comprises the highly conserved core sequence 5-GGA(A/T)-3. We found that overexpression of PEA3 resulted in a 5.8 -fold increase in transactivity of FAK when using luciferase as reporter, much stronger than that of Ets-1, Ets-2 overexpression, indicating a specific effect of E1AF/PEA3 on the FAK promoter (Fig. 4A). The PEA3 binding site in the FAK promoter is critical for activation by PEA3 (Fig. 4). Nuclear extract from B16F10 cells formed stronger band with the FAK promoter than that from B16F1 cells (Fig. 4F). Increased FAK mRNA levels were also found in highly metastatic B16F10 cells (Fig. 1). All these results suggested that PEA3 bound to the upstream region with specificity and transactivity in FAK promoter bearing PEA3-responsive element. To the best of our knowledge, this is the first evidence associating PEA3 transcription factors and FAK in melanoma metastasis.

**Figure 9:**
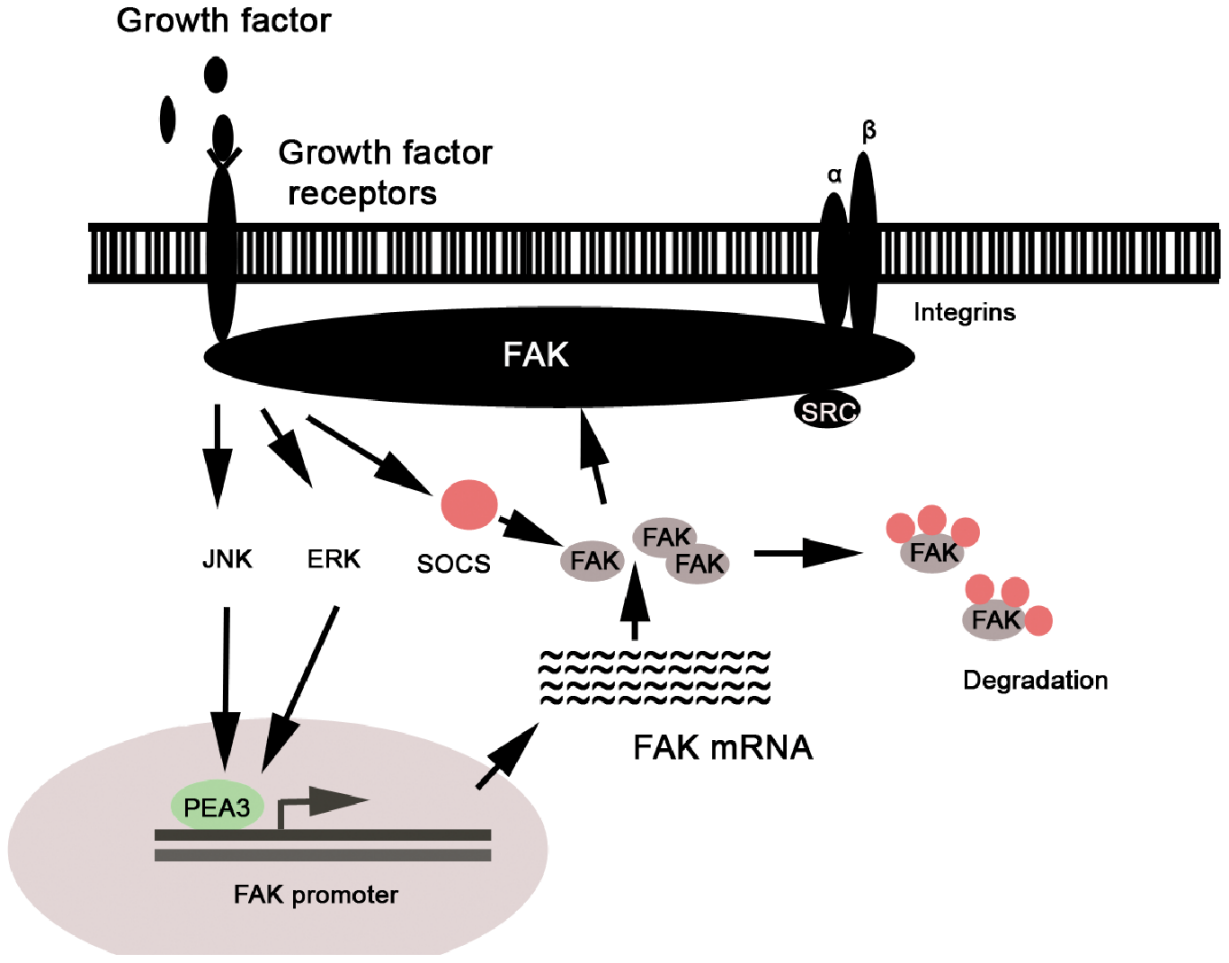
Schematic diagram of signal pathways underlying PEA3-mediated FAK gene transcription activation in melanoma tumor cells.

The E1AF/PEA3 was reported to be activated by Ras-MAP kinase signaling [40]. Consistent with these reports, we found ERK and JNK were also greatly involved in the transcriptional activation of FAK gene. FAK protein, in turn, can also stimulate Ras, ERK, JNK pathways in tumor cells [41]–[42]. Therefore, we hypothesis that ERK, JNK, PEA3 and FAK could form an amplification loop in metastatic tumor cells (Fig. 9), which probably facilitates metastatic cells to timely recruit more FAK molecules to function. Indeed, spatial and temporal FAK activity is usually required in the different steps of tumorigenesis and metastasis. Furthermore, even under the survival signaling by integrins and growth factor receptors, SOCS family proteins, like SOCS-1 or SOCS-3 that negatively regulated FAK activity could be upregulated to promote FAK degradation via proteasome-mediated pathway [43]. Thus, PEA3-mediated transcriptional upregulation of FAK may represent a novel regulatory mechanism for integrins or growth factors to deliberately control the progression of tumor growth or metastasis.

## Supporting Information

Figure S1 **Genomic organization covering the leader sequences of mouse FAK gene.** Exons −1, −2, −3, −4, −2 aM and −2 bM has been previously annotated and the supposed mouse promoter is indicated. Exon −5 was the newly discovered first exon by 5′-RACE analysis.
(TIF)

Table S1 **Patient characteristics (n = 100).**
(DOCX)

Table S2 **Correlation between PEA3 and FAK expression in 100 human primary oral squamous cell carcinoma specimens.** Overall, 77% (48/62) of the PEA3-positive cases from the 100 patients were FAK-positive. 92% (35/38) of the PEA3-negative cases from the 100 patients were FAK-negative.
(DOC)
