# Supplementary figures and images for: Requirement of PEA3 for Transcriptional Activation of FAK Gene in Tumor Metastasis
Source: PLoS One. 2013 Nov 18;8(11):e79336. doi: 10.1371/journal.pone.0079336 (PMC3832605; doi:10.1371/journal.pone.0079336)

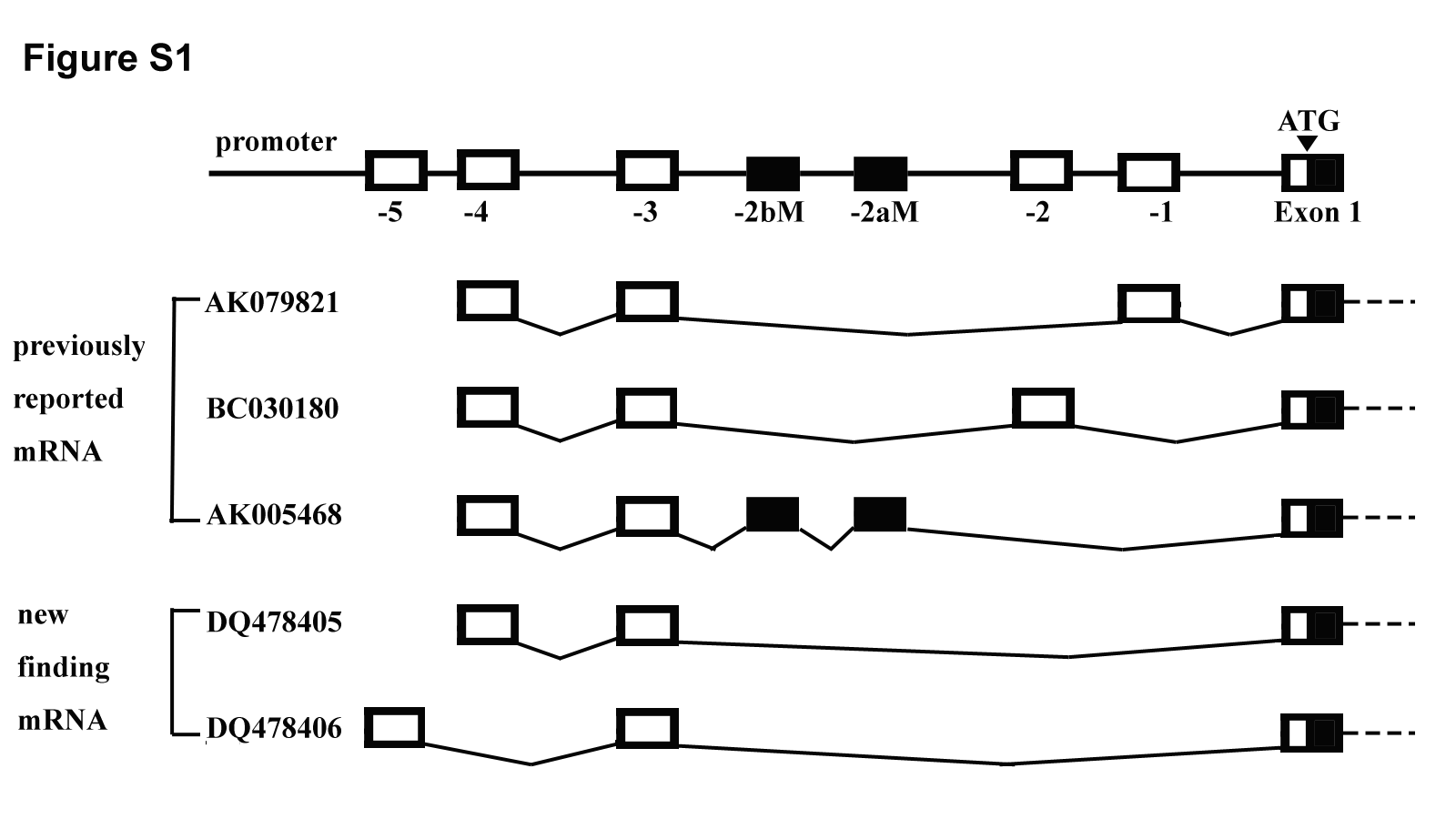

Supplement: Figure S1 — Genomic organization covering the leader sequences of mouse FAK gene. Exons −1, −2, −3, −4, −2 aM and −2 bM has been previously annotated and the supposed mouse promoter is indicated. Exon −5 was the newly discovered first exon by 5′-RACE analysis. (TIF) [file pone.0079336.s001.tif]
