# Supplementary material for: Requirement of PEA3 for Transcriptional Activation of FAK Gene in Tumor Metastasis
Source: PLoS One. 2013 Nov 18;8(11):e79336. doi: 10.1371/journal.pone.0079336 (PMC3832605; doi:10.1371/journal.pone.0079336)
Supplement: Table S1 — Patient characteristics (n = 100). (DOCX) [file pone.0079336.s002.docx]

**Table S1. Patient characteristics（n=100）**

| Clinical and pathological factors | | Number | FAK | |  |
| --- | --- | --- | --- | --- | --- |
|  |  |  | + | - |  |
| Ethnicity | Asian Chinese | 100 | 51 | 49 |  |
| Gender | Male | 54 | 27 | 27 |  |
|  | Female | 46 | 24 | 22 |  |
| Age(20-85) | <60 | 70 | 36 | 34 |  |
|  | ≥60 | 30 | 15 | 15 |  |
| Histologic type | well differentiated | 42 | 17 | 25 |  |
|  | Moderately differentiated | 58 | 34 | 24 |  |
| Lymph node  metastasis | Nonmetastasis | 57 | 20 | 37 | P<0.01 |
|  | metastasis | 43 | 31 | 12 |  |
| TNM stage  I | I/II (I: 26; II:29) | 55 | 23 | 32 | P<0.05 |
|  | III/IV (III: 21; IV: 24) | 45 | 28 | 17 |  |
