# Supplementary material for: Requirement of PEA3 for Transcriptional Activation of FAK Gene in Tumor Metastasis
Source: PLoS One. 2013 Nov 18;8(11):e79336. doi: 10.1371/journal.pone.0079336 (PMC3832605; doi:10.1371/journal.pone.0079336)
Supplement: Table S2 — Correlation between PEA3 and FAK expression in 100 human primary oral squamous cell carcinoma specimens. Overall, 77% (48/62) of the PEA3-positive cases from the 100 patients were FAK-positive. 92% (35/38) of the PEA3-negative cases from the 100 patients were FAK-negative. (DOC) [file pone.0079336.s003.doc]

**Table S2. Correlation between PEA3 and FAK expression in 100 human primary oral squamous cell carcinoma specimens.** Overall, 77% (48/62) of the PEA3-positive cases from the 100 patients were FAK-positive. 92% (35/38) of the PEA3-negative cases from the 100 patients were FAK-negative.

|  | FAK staining | | | |
| --- | --- | --- | --- | --- |
| PEA3 staining | Positive | Negative | Total | p value |
| Positive | 48 | 14 | 62 | P<0.01 |
| Negative | 3 | 35 | 38 |  |
| Total | 51 | 49 | 100 |  |
